# Emergency laparoscopic repair of coitus-induced vaginal cuff dehiscence: a case report

**DOI:** 10.1186/s13256-020-02362-4

**Published:** 2020-03-09

**Authors:** Ajay Agrawal, Kuan-Gen Huang, Marie Christine Valerie Mendoza

**Affiliations:** 1 Department of Obstetrics and Gynecology, BP Koirala Institute of Health Sciences, Dharan-17, Sunsari, State No-1, Sunsari, Nepal; 2 Department of Obstetrics and Gynecology, Chang Gung Memorial Hospital at Linkou, Kweishan, Taoyuan, Taiwan; 3 Chang Gung University College of Medicine, Kweishan, Taoyuan, Taiwan; 4 Department of Obstetrics and Gynecology, University of the Philippines - Philippine General Hospital, Manila, Philippines

**Keywords:** Complication, Minimal invasive surgery, Vaginal cuff dehiscence

## Abstract

**Background:**

Vaginal cuff dehiscence is a rare but potentially grave complication after total hysterectomy. Abdominal or pelvic contents are at risk of evisceration through the vaginal opening. It is associated with significant risk for patient morbidity, such as peritonitis, bowel injury, and sepsis.

**Case presentation:**

We report a case of vaginal cuff dehiscence in a 45-year-old multiparous Taiwanese woman who had undergone abdominal total hysterectomy and presented with vaginal cuff dehiscence precipitated by sexual intercourse. Immediate laparoscopic repair was done. Few authors have reported the utilization of the laparoscopic approach. It allows thorough inspection, visualization, and irrigation of the abdominal cavity. It is also associated with fewer intraoperative and postoperative complications.

**Conclusion:**

Laparoscopic repair is a safe treatment option to manage vaginal cuff dehiscence after total hysterectomy.

## Introduction

Vaginal cuff dehiscence (VCD) is a rare and distinct complication following pelvic surgery and has been reported with different modes of hysterectomy [1, 2]. The incidence has been reported to be less than 1% (0.24–0.31%) in large institutional studies [3, 4]. Its incidence varies according to the mode of hysterectomy, surgical technique, suture utilized for cuff closure, and operator’s experience. An observational study reported the occurrence to be highest for laparoscopic hysterectomy compared with total vaginal and total abdominal approaches [1]. The use of electrosurgery, particularly monopolar, and reduction of tissue margin during cuff repair may be contributing factors.

Post-hysterectomy VCD may present as early as 3 days to months or years after surgery with pelvic pain (60–100%), vaginal bleeding (30–60%), vaginal discharge (30%), or vaginal pressure/mass (30%) [1, 5, 6]. There is no consensus regarding the timing and mode of repair of cuff dehiscence. Possible approaches may be transabdominal, transvaginal, laparoscopic, or robot-assisted. We report a case of VCD in a multiparous woman who had undergone abdominal total hysterectomy and presented with VCD precipitated by sexual intercourse. Consent was taken from the patient for the purpose of this case report. The Ethical committee of Chang Gung Memorial Hospital, Linkou, Taiwan ruled out the need for ethical clearance for this case report.

## Case presentation

A 45-year-old multiparous Taiwanese woman had undergone abdominal total hysterectomy for adenomyosis and endometrioma at a local hospital 53 days prior to admission. Details of the surgery were not known. She had an uncomplicated postoperative course. One day prior to admission at Chang Gung Memorial Hospital, Linkou, Taiwan, she experienced lower abdominal pain and vaginal discharge after sexual intercourse. There was no history of fever, chills, dysuria, and fecaloid discharge from her vagina. She was evaluated at our emergency department where a pelvic examination showed bowel content protruding into her vagina with some purulent discharge. Her general condition was stable with normal results of hematology and biochemistry tests. With the clinical impression of complete VCD with cuff wound infection, she was planned for laparoscopic surgery.

**Fig. 1:**
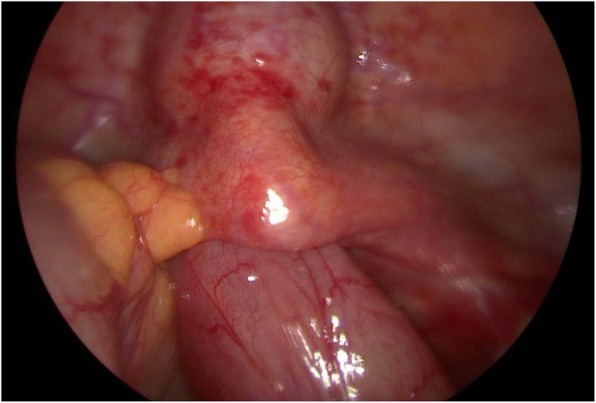
Loop of ileum protruding through the cuff

A standard four-port laparoscopy was performed with the umbilicus as the primary entry site using an 11 mm primary port and three 5 mm lateral accessory ports. On intraoperative examination, there were moderate adhesions between the left pelvic side wall and colon. There was a short loop of ileum protruding through the cuff which looked viable with no signs of ischemia or perforation (Fig. 1).

**Fig. 2:**
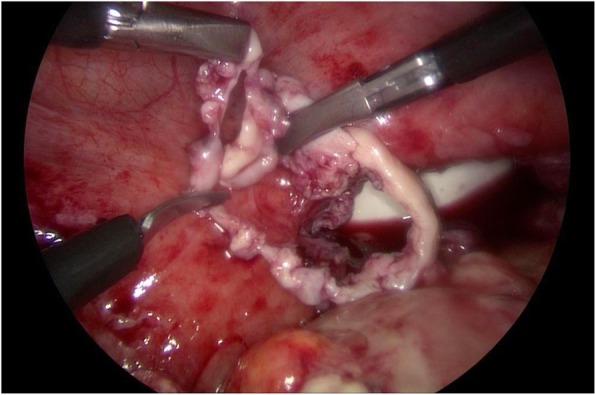
Excised vaginal cuff margin

After a thorough survey of her peritoneal cavity, adhesiolysis was done and the loop was carefully extracted from the cuff using atraumatic graspers. There were pockets of pus collection in the cul-de-sac that were cleared after adhesiolysis of the bowel loops. Complete dehiscence of the vaginal cuff was noted measuring approximately 5 cm. The cuff margins appeared inflamed. Careful downward dissection of her bladder and posterior peritoneum was carried out to achieve an adequate margin of vaginal cuff. Approximately 0.5 cm of cuff margin was excised circumferentially leaving viable tissue for proper suturing (Fig. 2).

The vaginal cuff was closed with a running Monocryl suture number 1–0 (poliglecaprone 25) with a depth of 1 cm vaginal cuff margin. Copious irrigation of her abdominopelvic cavity with saline solution was performed. A Jackson-Pratt drain was placed. Her postoperative course was uneventful. Intravenously administered antibiotics were completed and the drain was removed on the fifth postoperative day. She was followed up 2 weeks after discharge from our hospital: her pelvic examination revealed that the vaginal cuff was healing well. At a 1-month follow-up, her vaginal cuff was completely healed.

## Discussion and conclusions

VCD has been reported to occur in < 1% of patients post-hysterectomy. It can lead to serious and life-threatening complications when it is misdiagnosed or neglected. Hence, early recognition and prompt intervention are imperative. There is no standard method for surgical management of VCD. There are many reports of vaginal and abdominal repair of VCD, but few of these procedures are performed laparoscopically. We presented a case of VCD occurring after abdominal hysterectomy repaired by laparoscopy.

Although cuff dehiscence is a rare event, it may be triggered by factors such as postoperative cuff infection, early sexual intercourse, cigarette smoking, poor nutrition, obesity, menopausal status, and conditions that impair wound healing. In this report, coitus was the precipitating event for VCD which Hur *et al.* reported as the most common trigger [1]. Cuff cellulitis was also noted during laparoscopy with findings of cuff inflammation and pus collection at the posterior peritoneal pouch. In a study of case reports, the mean time to VCD was found to be 7 weeks for patients who had a total laparoscopic hysterectomy compared to 13 weeks for patients who underwent total abdominal hysterectomy (*p* = 0.01) [7]. Our patient presented at 7–8 weeks following abdominal hysterectomy. This complication can present during the early postoperative course or may extend to years following the surgery.

Consensus regarding mode of repair of VCD is not standardized. It can be repaired vaginally, abdominally, laparoscopically, or through a combined approach. The vaginal approach is minimally invasive but precludes observation of the entire abdominal cavity and irrigation of probable abscess. The choice for the mode of repair should be capably tailored to the patient’s presentation, bowel viability, surgeon’s judgement and expertise, and ability to obtain the best visualization of the dehiscence and entire abdominopelvic cavity.

Cronin *et al.* performed a review of original research, case reports, and case series published in the past 30 years on VCD [8]. Results showed that 51% of dehiscences were repaired vaginally, 32% were repaired abdominally, 5% were allowed to heal by secondary intention, and only 2% were repaired laparoscopically. Laparoscopic repair reduces the chances of operative injury to eviscerated bowel loop, which can occur during vaginal repair. It allows thorough inspection of the abdominal cavity and entire bowels. Early laparoscopic repair prevents complications such as ascending peritonitis and bowel ischemic injury, and recurrence of cuff dehiscence. It allows assurance of a good vaginal length as vaginal tissue may slough out due to long-standing infection. Furthermore, patient satisfaction is greater compared to the other approaches. With the laparoscopic approach, reopening of the previous abdominal wound was prevented along with its possible morbidity.

Use of antibacterial monofilament absorbable suture facilitates vaginal stump closure in laparoscopic hysterectomy without increasing the complications, such as cuff dehiscence, especially in less experienced operators [9].

The minimally invasive surgery approach leads to fewer intraoperative and postoperative complications, reduced postoperative pain, shorter hospital stay, and faster recovery. The use of laparoscopy for VCD has also been reported in the case of VCD during chemoradiation in a patient with endometrial cancer who had laparoscopic staging [10]. A case report that included a systematic review of VCD found only 3 cases (2.6%) using laparoscopy out of 116 cases reported [11]. One limitation to the use of this approach may be the need for notable technical expertise by an experienced surgeon. Hence, the mode of treatment should be carefully selected.

To reduce the incidence of VCD, it is recommended that patients avoid vaginal intercourse with deep penetration for at least 8 weeks postoperatively.

Laparoscopy is a feasible treatment option for the repair of VCD. It allows for a good vaginal margin for better healing, adequate peritoneal irrigation, and avoids the risk of bowel injury and morbidity associated with repeat laparotomy.

## Data Availability

Yes it is available.

## Abbreviation

VCD: Vaginal cuff dehiscence
